# Olfactory Identification as a Biomarker for Cognitive Impairment: Insights from Healthy Aging, Subjective Cognitive Decline, and Mild Cognitive Impairment

**DOI:** 10.3390/ejihpe14120196

**Published:** 2024-11-29

**Authors:** Jaime Bouhaben, Alice Helena Delgado-Lima, María Luisa Delgado-Losada

**Affiliations:** Experimental Psychology, Cognitive Processes and Speech Therapy Department, Faculty of Psychology, Complutense University of Madrid, 28223 Pozuelo de Alarcón, Spain; jaimebou@ucm.es (J.B.); alicedel@ucm.es (A.H.D.-L.)

**Keywords:** olfaction, neuroplasticity, aging, subjective cognitive decline, cognitive impairment, mild cognitive impairment, olfactory identification, biomarker

## Abstract

**Introduction**: This study aims to investigate the relationship between olfactory identification (OI) and cognitive impairment by examining OI abilities across various stages of cognitive deterioration. **Methods**: A total of 264 participants were divided into three groups based on cognitive status: cognitively healthy, subjective cognitive, and mild cognitive impairment. All participants were assessed using the Sniffin’ Sticks Olfactory Identification test and a comprehensive neuropsychological test battery. **Results**: Our results highlight the main effects of age and cognitive status on OI scores. Regarding cognitive abilities, OI is associated with measures of short-term memory, long-term, working memory, and selective attention. Finally, logistic regression models showed that OI is a significant predictor for discriminating SCD from CH, MCI from CH, and MCI from SCD. **Discussion**: These findings suggest the addition of olfactory identification measures in neuropsychological assessments could improve the early detection of individuals at risk for cognitive impairment.

## 1. Introduction

Cognitive impairment in aging is a physiological process linked to age, which, in some cases, can lead to pathology up to dementia. Within this slow and continuous process of deterioration, two transition phases can be distinguished. Subjective cognitive complaints (SCCs) are defined as concerns about the worsening or malfunctioning of some aspect of cognition, regardless of whether the cognitive impairment is detected by standardized objective tests [1]. Then, the second corresponds to mild cognitive impairment (MCI) in which the person presents a cognitive impairment that does not interfere or minimally interferes with the activities of daily living [2].

Population studies with follow-ups from 3 to 20 years report that SCCs may predict MCI and/or dementia, usually in combination with other variables such as being a woman or having a high educational level [3,4] and being a carrier of the APOE e4 gene [5], causing concern in the patient [6,7,8]. Patients who manifest these cognitive complaints are more likely to progress to MCI and dementia [6,7,8,9]. Results from longitudinal studies with clinical samples are more contradictory. On one side, memory complaints do not often predict progression to dementia [10,11]. However, other studies do find an increased risk of progression to dementia in participants with SCC [11]. The presence of SCC supposes a criterion for subjective cognitive decline (SCD) [6,7]. SCD increases the risk of developing pathologies such as MCI and Alzheimer’s disease [6,7]. Some studies and meta-analyses point out that patients with SCD or subjective complaints are 1.73-2 times more likely to develop MCI or dementia [12,13,14,15,16].

In SCD, one should perceive a decline in cognitive performance in daily life with respect to a previous stage of subjectively intact cognition. This transition from normality to a stage of cognitive difficulties is not reflected in standardized tests, whose performance remains within a normal range for age and educational level [6,7]. Criteria also state that SCD is unrelated to an acute event and persists over time, so memory complaints must be present within the past 6 months. This is because it is believed that SCD can arise as a result of both preclinical AD and other non-neurodegenerative conditions. Thus, monitoring the course and progression may provide insight into the possible etiology [6,7,8].

The prevalence of SCD is estimated between 14% and 54% [17]. It is still unclear what factors moderate the progression between complaints and objective cognitive impairment [16]; therefore, the early identification of the progression from SCD to MCI is of great importance for public health [18].

Olfactory identification (OI), the capacity to recognize and name specific odors, is recognized to be impacted by aging [19,20,21,22], MCI [23,24,25], dementia [21,26,27], and more recently, it has been associated with an asymptomatic preclinical stage of AD, SCD [16,28,29,30].

A single measure of OI dysfunction might predict, not only an increase in cognitive decline up to 15 years later [31,32,33,34] but also a higher risk of conversion from normal cognition to MCI [35] and from MCI to AD [32]. Furthermore, olfactory deficits are associated with neurodegenerative findings detectable by neuroimaging [34,36,37,38].

Deterioration in OI is intimately related to cognitive decline in various pathological conditions and aging. The association between the neuroanatomy of the olfactory system and cognition [32,39,40] is well supported by the literature. This association highlights a close relationship with memory, given the proximity of the hippocampus and olfactory cortex. Additionally, the prefrontal cortex is also a neocortical region involved in olfactory processing, which suggests a relationship between OI performance and executive functioning. Studies with functional neuroimaging support the relevance of these regions in olfactory functioning [41]. Olfactory and cognitive performance share neural correlates affected by physiological aging. Brain structures closely related to the olfactory system show significant early histopathology in MCI and AD, and the brains of AD patients invariably show neuropathology in the entorhinal cortex [42,43]. It is worth highlighting a correlation between psychophysical measures of olfactory function and hippocampal volumetric loss [43,44]. Several studies have emphasized the potential usefulness of an olfactory identification test and hippocampal volume loss for the early detection of AD [45,46].

Olfactory status is reliably associated with cognitive health, and the severity of olfactory dysfunction appears to be associated with the rate of cognitive impairment [27,34,47,48,49].

The aim of this study is to understand the association between OI and cognitive impairment by analyzing the olfactory identification abilities across different stages of cognitive deterioration (CH, SCD, MCI) and determining if these variations can help categorize individuals accurately while exploring the relationship between OI and cognitive domains (memory, executive functioning, attention, executive functioning, visuospatial skills, and language) in a sample of older adults representing a continuum of performance capability. Thereby, this study contributes to the broader goal of enhancing early diagnostic capabilities in cognitive impairment.

## 2. Materials and Methods

### 2.1. Participants

An initial pool of 297 Spanish-speaking older adults volunteered to participate in this study. All the participants were recruited through advertisements in senior centers and health centers in the Community of Madrid (Spain). All the participants were informed about the study guidelines and gave written informed consent to participate in this study.

The general inclusion criteria were (i) age ranging from 55 to 90 years; (ii) no prior diagnosis of dementia; (iii) no records of any neurological alterations, such as stroke, head trauma, or encephalitis; (iv) absence of current otorhinolaryngological issues; and (v) compliance with testing procedures. Once the general inclusion criteria for this study were fulfilled, the participants were assigned to one of three clinical cohorts, based on their cognitive status. These cohorts were cognitively healthy participants (CH), participants with subjective cognitive decline (SCD), and participants with mild cognitive impairment (MCI). A fourth clinical cohort of participants with a serious risk of dementia and/or probable Alzheimer’s disease (AD) was identified. However, due to its limited sample size, this cohort was excluded from further analyses. The inclusion criteria for the cognitively healthy (CH) cohort were screening score > 26 (MoCA > 26), normal performance in neuropsychological tests, and no self-reported complaints about their cognitive status. The diagnostic criteria for subjective cognitive decline were screening score > 26 (MoCA > 26), performance in neuropsychological tests within the normal limits, and self-reported complaints about their cognitive status. These complaints were measured with three questions: (Q1) *Do you have memory problems?*; (Q2) *Do you forget where you put things?* and (Q3) *Do you forget the names of family and acquaintances?* Response options for Q1 were *yes* or *no*. This is a frequently asked question in most population studies about SCD [50]. Both Q2 and Q3 have two alternatives: *Rarely* (interpreted as *no*) or *Frequently* (interpreted as *yes*). Those participants who answered *yes* to Q1, Q2, and Q3 were considered to satisfy the self-reported complaints criteria. These SCD criteria were obtained from Jessen et al. (2020) [7]. MCI diagnosis was performed following the criteria in Albert et al. (2013) [51]: (i) evidence of concerns about cognitive changes compared to a previous level, corroborated by an informant; (ii) evidence of worse than expected performance in at least one cognitive domain (−1.5 SD), according with age and educational background; (iii) preservation of functional independence in activities of daily life; and (iv) disagreement with criteria for diagnosis of dementia.

The exclusion criteria from the study were (i) medical history of olfactory alterations, including nasal polyps, rhinitis, rhinosinusitis, or previous otorhinolaryngological surgery; (ii) medication intake which may affect olfactory performance (such as some antibiotics, antiepileptics, antithyroids, benzodiazepines, or antiarrhythmics); (iii) presence or suspicion of psychiatric alterations, such as depressive or psychotic disorders (self-reported by the participant or present in clinical history); and (iv) presence of olfactory deficits or alterations due to COVID-19 infection (self-reported or present in the clinical history).

The final sample, after eligibility criteria, was composed of 264 participants (68.04 ± 7.58 years; 172 women of 67.4 ± 7.27 years and 92 men of 69.2 ± 8.05 years).

### 2.2. Study Design

The present study follows a cross-sectional non-experimental design, as no manipulation for independent variables nor random allocation was performed and measures were taken once per participant. The assessment procedure was carried out between September 2021 and September 2022. The complete procedure was administered in one visit. In this visit, all participants underwent the neuropsychological test battery described in Section 2.3 and the Olfactory Identification Test (from Sniffin’ Sticks Olfactory Test). Supplementary Table S1 displays a summary of the study methods.

This research followed the tenets of the Declaration of Helsinki (Edinburgh, 2013) and was approved by the Ethics Committee from University Hospital San Carlos (Madrid, Spain, internal codes 17/192-E and 18/422-E_BS). This study was adjusted to standards of good clinical practice (art.34 RD 223/2004; community directive 2001/20/CE) and to the protection of personal data and confidentiality (European Data Protection Regulation, and in accordance with the Organic Law 3/2018 on the Protection of Personal Data and Guarantee of Digital Rights).

### 2.3. Measures and Procedure

The assessment protocol comprised a screening test of general cognitive status, a questionnaire of cognitive complaints, an olfactory evaluation, and a battery of neuropsychological standardized measures to measure these cognitive domains: semantic memory, episodic memory, executive function, working memory, attention, and language.

- *Global cognitive status*: General cognitive performance was tested with the Spanish adaptation of the Montreal *Cognitive Assessment (MoCA)* instrument [52]. MoCA is a widely used screening tool that assesses several cognitive domains: attention, concentration, memory, language, calculation, orientation, and executive functions. Due to its nature as a screening tool, MoCA grants cutoff points for cognitive impairment, which may accurately guide its diagnosis. MoCA’s original publication points to 26 as the cutoff point between cognitive impairment and healthy aging [53]. This cutoff point was adopted in the present study to help with the classification of participants with MCI.
- *Olfactory performance*: Olfaction was assessed with the Spanish adaptation of the Identification Smell Test, from the Sniffin’ Sticks Olfactory Test. This adaptation was developed and validated by Delgado-Losada et al. (2020, 2021) [54,55]. In the Spanish version of this test, potential bias due to cultural aspects of odor descriptors was addressed. Recognition and subjective intensity scores were obtained for the present study. Both scores are part of Delgado-Losada et al.’s (2021) study [55]. *Recognition score*: This is the original score of the test. It represents whether each odorant is correctly identified through a four-alternative forced-choice method. The odorant is shown to the participant, and they have to identify the target odor between four odor descriptors. Correct answers from the 16 items are added in order to calculate this score. *Subjective intensity score*: This score intends to measure the subjective intensity of an odor. It was designed and validated by Delgado-Losada et al. (2021) [55]. After each trial, participants have to score the subjective intensity of the smell presented with a 1–10 visual analog scale (1 implies a minimal intensity and 10 implies an extraordinary intensity). This score provides an additional value to olfactory identification performance. The total score is calculated as the arithmetic mean of the 1 to 10 intensity given to each item.
- *California Verbal Learning Test-II (CVLT-II)* [56]: CVLT is a neuropsychological instrument that intends to measure short-term and long-term episodic memory through a wordlist task. Participants listen to a list of 16 words, with 1 s intervals, and they are asked to memorize as many as they are able to. Immediately after five trials, participants are asked to recall the target words (immediate recall). After 20 min, they are asked again to recall the words (delayed recall). Two scores were taken, the immediate recall score and the delayed recall score, both related to episodic memory.
- *Digit Span Test* from Wechsler Adult Intelligence Scales-IV (WAIS-IV) [57]: The test consists of two parts: the digit span forward (DSF) and the digit span backward (DSB). In DSF, a number is spoken each second in various sequences, and the individual is expected to repeat the numbers in the same order. In the DSB section, the individual is asked to repeat the sequences of numbers in reverse order. In both sections, the length of the number of sequences gradually increases. There are two trials for each digit span. Two scores were obtained, including the DSF span score, which measures short-term memory, and the DSB span score, which measures working memory.
- *Cancellation Test.* This is a classic cancellation task, adapted from the ELSA study (https://www.elsa-project.ac.uk; accessed on 10 November 2024) (Huppert et al., 2004) [58] and implemented in the ELES project [59], which measures selective attention and processing speed. The task consists of a matrix of random letters distributed in rows and columns. Participants are asked to mark target letters (P and W) as fast as possible in 1 min. The number of correct (target letters correctly marked) and incorrect answers (omission errors) were considered.
- *Rey-Osterrieth Complex Figure Test (RCFT).* This is also an instrument widely administered in clinical neuropsychology to measure visuospatial skills and non-verbal memory [60]. Participants are exposed to a complex figure they have to copy on paper and memorize. After the first copy, they are asked to repeat the figure immediately (immediate recall). Then, after 20 min, participants are asked to draw the figure again (delayed recall). Total scores from immediate recall and delayed recall were obtained.
- *Verbal Fluency Test*: Verbal fluency measures are often included in neuropsychological protocols to assess language and executive functioning in both cognitively healthy and cognitively impaired conditions. According to Lezak et al. (2012) [61], participants have to verbally declare as many words as they are able to, following a certain condition, in 1 min per condition. In this study, two conditions were administered: phonological (generate as many words starting with the letter F as possible in one minute) and semantic (name as many animals as possible in 1 min). The total score per condition was obtained, as intended measures of phonological and semantic verbal fluency, respectively.

Vocabulary Test, from Wechsler Adult Intelligence Scales-IV [57]. This is a test in which participants are asked to explain and define a concept from its word, starting with easy words that get progressively more difficult. It is usually administered to measure verbal comprehension, crystallized intelligence, lexical knowledge, and the ability to retrieve information. The total score was obtained as a measure of premorbid intelligence and cognitive ability.

Trail Making Test. This is a classic neuropsychological task that aims to measure executive functioning. Two parts were administered [62]. TMT-A consists of a piece of paper composed of 25 randomly distributed circles, numbered from 1 to 25. Participants have to connect them with a constant line, in ascending order, as quickly as possible, but maintaining accuracy in order. TMT-A mainly assesses visuospatial processing speed and sustained attention. Similarly, TMT-B also consists of a piece of paper with randomly distributed circles, but in this case, those circles contain numbers from 1 to 13 and letters from A to L. Participants have to connect them in ascending order, intercalating numbers and letters (1-A-2-B-3-C and so on). TMT-B mainly assesses cognitive flexibility and the ability to alter between two sets of stimuli. Two scores were obtained, including the time of resolution (in seconds) of parts A and B.

### 2.4. Statistical Analyses

**Figure 1:**
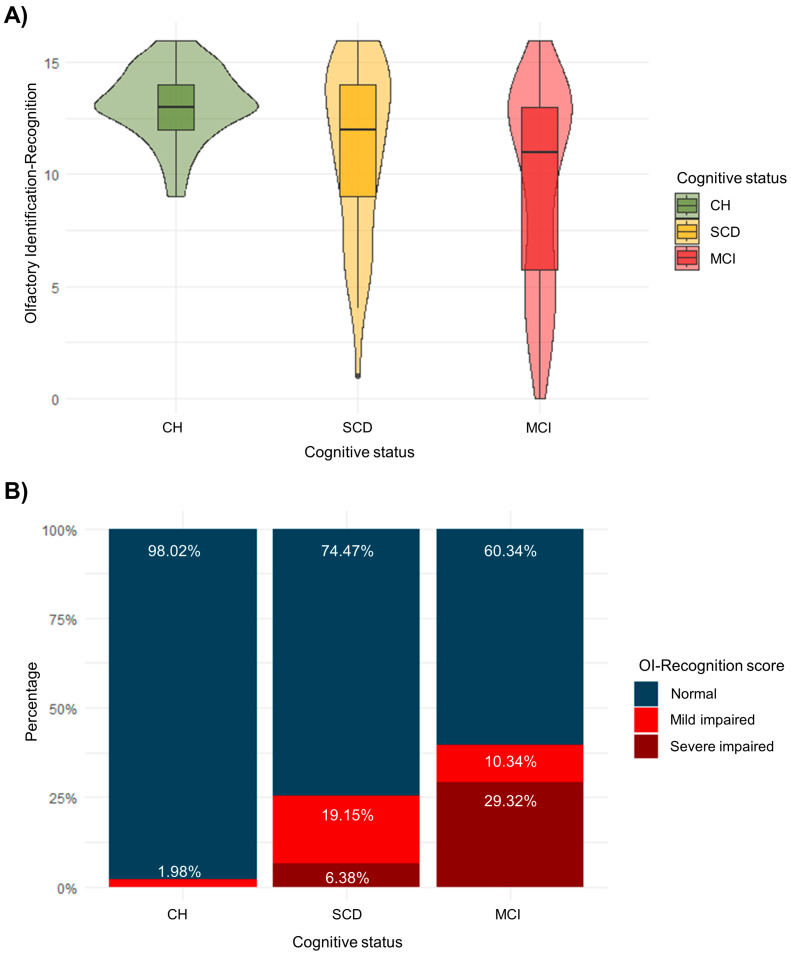
(**A**) Violin and boxplot for each cognitive group. (**B**) Distribution of normal, mild impaired, and severe impaired olfaction per cognitive group.

The entire statistical analysis plan was carried out with R software, version 4.3.0 [63]. In the first place, a descriptive analysis was conducted. The mean and standard deviation were calculated for each cognitive status group, as well as chi-squared tests for dichotomous variables and F tests for continuous variables. In addition, percentiles 10 and 5 of the Olfactory Identification-Recognition score were used to describe the participants’ olfactory performance as normosmic (>10th percentile), mildly impaired (<10th percentile), and severely impaired (<5th percentile) in Figure 1B [54,55].

Next, a two-way between-subject ANOVA model with Type III sum of squares was adjusted for the Olfactory Identification-Recognition score as a dependent variable, with cognitive status (*CH*, *SCD,* and *MCI*), age, and the interaction cognitive status × age as factors. Age categories were established as [<60, 60), [60, 70) and [70, >70). Post hoc between-group multiple comparisons were conducted under Tukey’s HSD test, with adjusted p values due to multiple comparisons.

After that, relationships between cognitive measures and olfactory performance were studied. Linear regression models were computed for each cognitive measure, with age and olfactory identification as predictors. A stepwise method was applied to identify the contribution of the Olfactory Identification-Recognition score for each cognitive variable: a baseline model (model 1) was firstly computed with *age + sex* as predictors. Model 1 was compared to model 2, which also includes olfactory performance. Regarding sex, due to variable coding, positive estimates favor women, whereas negative estimates favor men. Alpha was set at α = 0.0038 under Bonferroni correction (α = 0.05/13).

Finally, logistic regression models were conducted in order to categorize the participants into their respective cognitive groups (CH vs. SCD, CH vs. MCI, and SCD vs. MCI). This analysis intended to find how the olfactory identification score is able to help to categorize the participants into their respective cognitive status groups, and hence, the potential clinical use of this test. For each comparison (CH vs. SCD, CH vs. MCI, and SCD vs. MCI), two models were calculated, including a baseline model (model 1) that classifies *age + sex* as predictors, and its performance was compared to model 2, which was computed with age, sex, and the Olfactory Identification-Recognition score as predictors. This method was taken from Delgado-Lima et al., 2023 [9]. In these models, positive estimates also favor women and negative ones favor men. For this analysis, alpha was set at α = 0.017, under Bonferroni correction (α = 0.05/3).

## 3. Results

A descriptive analysis of the sample by cognitive status is displayed in Table 1. The effect of *age* is statistically different from 0, due to the mean age in the MCI group being lower. Hence, this effect was considered in subsequent analyses. However, no differences either in sex or frequent alcohol consumption were elucidated. Regarding the Olfactory Identification-Subjective intensity score, there is no evidence of differences between cognitive groups. Consequently, this score was not used in further analyses. Figure 1A shows distributions for each cognitive group with violin plots, whereas Figure 1B reports the percentages of normal, mildly impaired, and severely impaired olfactory identification per cognitive group.

**Figure 2:**
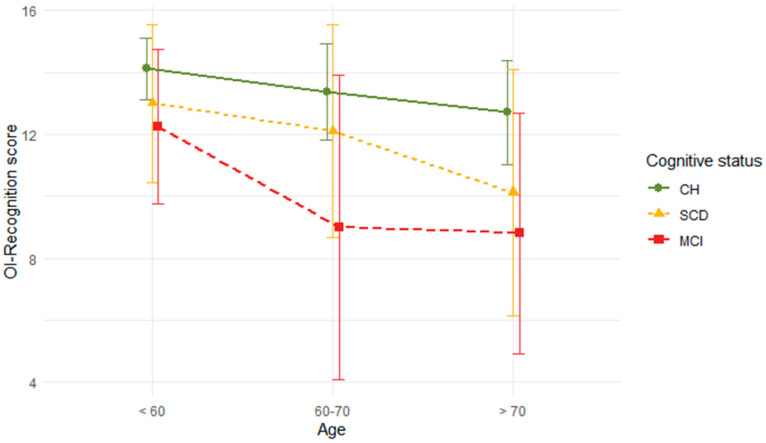
Mean graph of the Olfactory Identification-Recognition score by age and cognitive status.

The two-way ANOVA model on the Olfactory Identification-Recognition score shows the main effects of *age* (*F* = 36.52, df = 2, *p* < 0.0001) and *cognitive status* (*F* = 33.65, df = 2, *p* < 0.0001). There is no evidence to support the *cognitive status* × *age* interaction effect (*F* = 0.825, df = 3, *p* = 0.48). The mean graph is shown in Figure 2. Post hoc comparisons on *age* show, on one side, significant differences between [<60, 60) and [70, >70) (dif = 2.151, *p* = 0.0016) and between [60, 70) and [80, >80) (dif = 2.302, *p* <= 0.0005). Both differences favor the younger category. On the other side, post hoc comparisons on *cognitive status* report significant differences between the three cohorts, with CH participants scoring higher and MCI participants scoring lower: CH and SCD (dif = 1.801, *p* = 0.007), SCD and MCI (dif = 1.847, *p* = 0.005), and CH and MCI (dif = 3.647, *p* < 0.0001).

Linear regression models for each cognitive variable are reported in Table 2. In each case, model 1 predicts cognitive performance with *age*. The stepwise introduction of the Olfactory Identification-Recognition score in model 2 was tested with R^2^ and with an F-test comparing both models. These results highlight the effect of olfactory identification performance in measures of short-term memory (CVLT—immediate recall score, b = 0.589, SE = 0.112, *p* < 0.0001; DSF, b = 0.09, SE = 0.023, *p* = 0.0001; Rey Complex Figure Test—immediate recall score, b = 0.542, SE = 0.164, *p* = 0.001), long-term memory (CVLT—delayed recall score, b = 0.254, SE = 0.066, *p* < 0.0001), semantic memory (semantic verbal fluency—total score, b = 0.391, SE = 0.089, *p* < 0.0001), working memory (DSI, b = 0.098, SE = 0.025, *p* = 0.0001), and selective attention (Cancelation—correct answers, b = 0.309, SE = 0.103, *p* = 0.003). Olfactory identification also relates to cognitive performance in the Vocabulary Test—total score (b = 0.577, SE = 0.248, *p* = 0.021) and TMT B—seconds (b = −4.258, SE = 1.519, *p* = 0.005), but these effects are not adjusted for multiple comparisons (*p* < 0.0038). Hence, these effects should be interpreted carefully. Sex also seems to be a statistically significant predictor of the Vocabulary Test—total score (b = −4.338, SE = 1.525, *p* = 0.005). The negative association implies that men tend to score higher in this task. However, this effect is not adjusted to corrected *p* < 0.0038 either, so the interpretation should be careful.

Finally, the discrimination power of olfactory identification to differentiate the cognitively healthy participants from the participants with subjective cognitive decline (CH vs. SCD) and the participants with mild cognitive impairment (CH vs. MCI) was tested with logistic regression models. The results are displayed in Table 3, Table 4 and Table 5. In each table, model 1 is the baseline model, as it shows performance with *age* and *sex* as the unique predictors. Right after model 1, model 2 implies the introduction of the Olfactory Identification-Recognition score for analysis if the model improves. A comparison between model 1 (baseline) and model 2 was performed with ANOVA with the likelihood ratio test.

**Figure 3:**
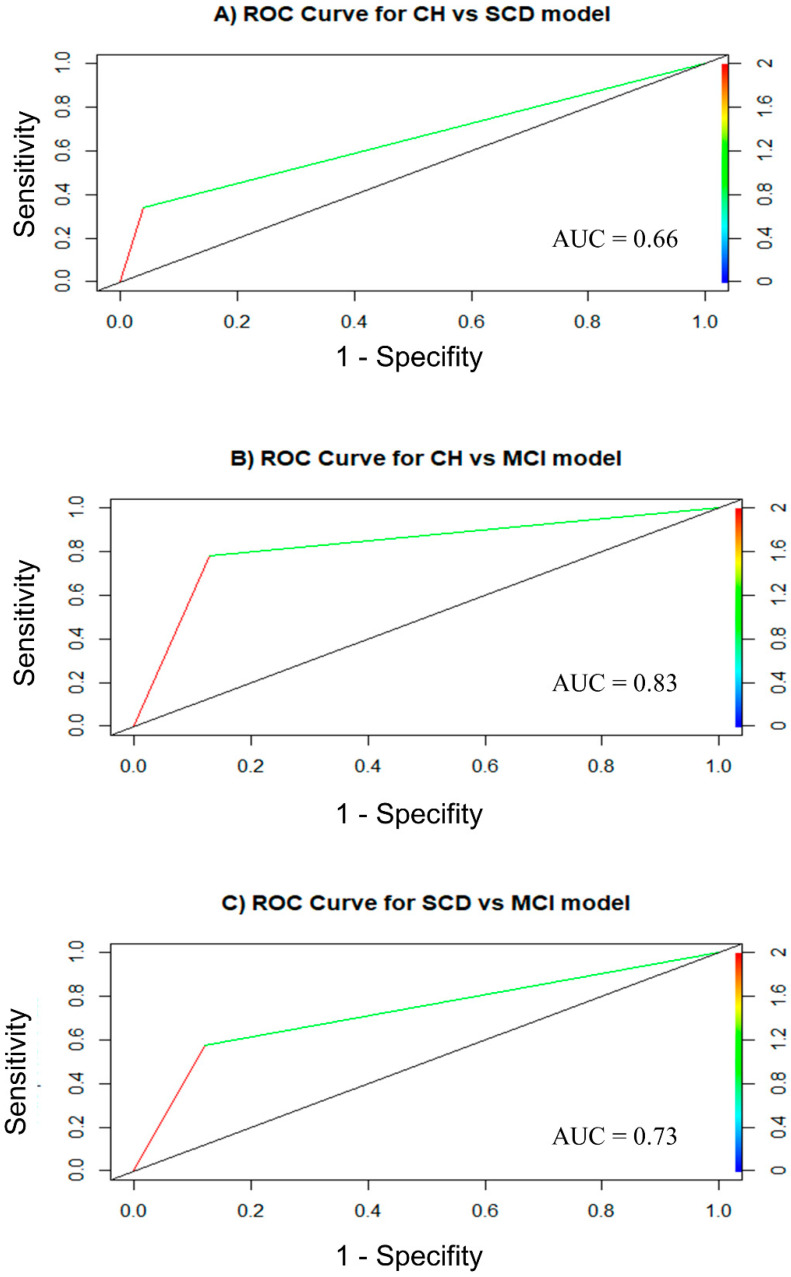
ROC curves for: (**A**) CH vs. SCD, (**B**) CH vs. MCI, and (**C**) SCD vs. MCI.

The results for the CH vs. SCD classification are shown in Table 3. In this first case, model 1 is a null model, with no effect of *age* (*p* = 0.572) or *sex* (*p* = 0.186). However, the addition of the Olfactory Identification-Recognition score significantly improves the logistic model (model 1 vs. model 2, chi = 17.952, df = 1, *p* < 0.0001). Next, the results for the CH vs. MCI comparison are reported in Table 4. Again, model 1 is a null model, with no statistically significant predictor, as the effect of age and sex are not statistically different from 0 (*p* = 0.086 and *p* = 0.746, respectively). Similar to the CH vs. SCD model, the addition of the Olfactory Identification-Recognition score significantly improves the classification power of model 2 (model 1 vs. model 2, chi = 124.06, df = 1, *p* < 0.0001). Plus, with the addition of the OI score, the effect of age is now statistically significant. Nevertheless, there is no evidence to assume the effect of sex. Finally, the results for the CH vs. MCI comparison are displayed in Table 5. The inclusion of the Olfactory Identification-Recognition score in model 2 also improves its classification power (model 1 vs. model 2, chi = 49.782, df = 1, *p* < 0.0001). In model 2, age is also a significant predictor (*p* = 0.0009), whereas sex cannot be considered statistically significant (*p* = 0.054). ROC curves for models with the Olfactory Identification-Recognition score are reported in Figure 3.

## 4. Discussion

In the present study, the association between OI dysfunction and the risk of cognitive impairment (SCD and MCI) was analyzed. OI tests are frequently employed to assess olfactory function in individuals with MCI and dementia [64,65,66].

We found that OI differed significantly between CH, SCD, and MCI. Across the continuum of cognitive risk, OI decreases in a stepwise manner, as the participants with MCI performed lower than SCD, and SCD performed lower than CH. Our results indicated that 40% of the participants with MCI had OI dysfunction (Figure 1B). Different investigations indicate that sensory impairments, such as OI deficits, may precede cognitive impairment, and several years before symptoms of cognitive impairment manifest in AD, individuals may already exhibit compromised OI abilities [67,68,69]. The literature is unanimous in its interest in including OI analysis in MCI and SCD groups, whose results are along the same lines as ours, observing a worse performance in OI in the MCI group compared to SCD and in SCD compared to CH [70,71,72].

Our results are in line with many other authors who have found a pattern of deterioration in OI test performance similar to ours between CH participants and SCD participants [28,29,73]. This trend was also supported by the meta-analysis by Jobin et al. (2021) [48]. Their results noted that individuals with SCD have lower performance on OI tests compared to cognitively healthy (CH) older adults. Moreover, Sohrabi et al. (2009) also found statistically significant differences in OI between CH and SCD cohorts [74]. Therefore, the differences in olfaction between CH and SCD groups seem to be consistent.

In our study, ANOVA analysis on olfactory identification revealed significant main effects for both age and cognitive status. The significant effect of age on olfactory performance is consistent with prior research, indicating that olfactory performance decreases with age. This deterioration is attributed to various factors, including the degeneration of olfactory receptor neurons, changes in the olfactory bulb, and alterations in central processing regions [19,75,76]. This association reflects age-related physiological changes in the central nervous system, which may affect both olfaction and cognitive function [77].

Promising and consistent results about how OI alterations alone predict the progression to AD from its asymptomatic preclinical stage (including MCI and even earlier stages such as SCD) have been reported [78,79]. However, our results suggest that combining cognitive assessments with an olfactory identification measure could improve the early detection of at-risk individuals. Olfactory deficits have been shown to precede cognitive symptoms and correlate with the severity of cognitive impairment. Thus, like other authors, we can point out that the odor identification test scores are influenced by non-olfactory cognitive abilities [39,80,81].

Likewise, when comparing the cognitive status across the three samples, CH scored significantly higher than those with SCD and those with MCI. Similarly, the SCD participants demonstrated better cognitive outcomes compared to the MCI participants. These results align with the existing literature, which consistently shows that cognitive performance declines progressively from CH to SCD and then to MCI [7,15,16,18,82]. Additionally, the SCD participants demonstrated better cognitive outcomes compared to the MCI participants, further supporting the notion that SCD represents an asymptomatic preclinical stage in the continuum of cognitive decline.

When analyzing the results for each cognitive domain and their association with OI, the results indicate that a decrease in OI is associated with lower performance in episodic, semantic, and working memory, executive functions, and attention and processing speed, and there is no relationship with performance in language or visuospatial skills. The relationship between these cognitive domains and odor identification function might suggest overlapping structural and physiological disease processes in regions of the central nervous system related to olfactory factors [83]. Regarding sex differences, there are examples in the literature that support them in OI [76,84,85], so we considered that sex could be a potential covariate within linear and logistic regression models. Our results highlight that sex seems to be a statistically significant predictor for the vocabulary score, under uncorrected p-values. This effect favors men. However, we found no evidence of sex differences predicting any other cognitive score, when combining it with OI. These results regarding sex are in line with our previous studies [54,55].

In episodic memory, our results revealed that OI is related to both verbal episodic memory (measured with CVLT-II immediate and delayed recall scores) and visual episodic memory (measured with RCFT immediate recall score). In relation to semantic memory, OI is associated with the semantic verbal fluency score. There is consensus in indicating that, to a greater extent, in the semantic fluency condition, not in the phonological one, people with progressive cognitive impairment generate significantly fewer words [86,87]. In another meta-analysis by Jobin et al. [88], OI was related to episodic memory and semantic memory, although effect sizes were relatively small. In addition, our findings are in line with several studies in which a relationship was found between semantic memory deficits and lower performance on the OI test [31,70,89,90]. OI relies on prior semantic knowledge, the ability to access that knowledge, and the capacity to link it to linguistic labels, along with organization and information processing strategies. Vocabulary tests are related to this crystalized knowledge [91], so this could explain the results in the vocabulary scores and the potential sex differences found in this score. Difficulties at any level of semantic processing can affect task performance [92]. The entorhinal cortex, which connects with the hippocampus and is essential in the formation and recognition of memories, also plays a very important role in olfactory processing [93,94]. It is now thought that AD likely originates in the entorhinal cortex, a region essential for olfactory processing [28]. Thus, OI could be key in the early detection of AD, as these deficits may appear in individuals with SCD or suspected preclinical AD [73]. Apparent but milder deficits have been observed in at-risk individuals without dementia (i.e., individuals with APOE4 allele genotypes [95] and first-degree relatives of AD patients [96,97]).

In executive functions, our results indicate that OI is related to working memory, verbal fluency, and cognitive flexibility. In working memory, OI dysfunction is associated with lower scores on DSI scores [98]. As noted above, OI is associated with semantic verbal fluency (also an executive function measure). In relation to cognitive flexibility, OI might be related to TMT-B. TMT-B may indicate dysfunction in the dorsolateral prefrontal cortex, so our results suggest that processing in frontal regions is more important than olfactory processing. Our results support Challakere et al. (2022) [99], who found worse performance in TMT-B performance as cognitive impairment progresses. These results may suggest that orbitofrontal processing (an area thought to be relevant in olfactory processing) would be more affected than processing in other frontal regions (i.e., dorsolateral). However, we found no relationship between OI and performance in TMT-A, in disagreement with Uchida et al. (2020) [100].

Lastly, OI was found to be a powerful variable for classifying patients into CH, SCD, and MCI categories. The HC vs. SCD logistic regression model suggests that olfactory identification has a significant role in distinguishing between CH and SCD individuals. Similarly, the CH vs. MCI logistic regression model is also significant in distinguishing MCI from CH when including the OI score, in agreement with our previous findings [19]. Moreover, the present study also evidences that OI is a useful tool for distinguishing patients with MCI from those with just previous SCD. Nevertheless, there is no evidence in any model to assume that sex might be a predictive variable to distinguish CH, SCD, and MCI. Thus, our findings align with and extend those of previous studies. For instance, Wang et al. (2021) [29] found that OI scores were progressively lower from CH to SCD, MCI, and AD. Although OI measures are useful for the early detection of dementia, these are underutilized in clinical examinations. This could indicate a lack of familiarity with these instruments and uncertainty about how they integrate with a traditional neuropsychological assessment. Consequently, our findings may help practitioners by offering a framework for interpreting OI measures.

We consider that a strong point of our study is the application of a broad neuropsychological assessment protocol, which allowed us to deepen the relationship between OI and the different cognitive domains. The combination of the OI test and cognitive tests in various domains can improve the ability to predict future cognitive impairment in older adults without dementia [69]. Nevertheless, the strengths of the present study do not exempt it from some limitations. The main limitation lies in the study design. This is a cross-sectional study, so each cognitive group is composed of different participants. Hence, it is not possible to interpret statistical differences between groups as the development of dementia. Future research should explore the relationship between olfactory identification and cognitive performance using longitudinal data, allowing for conclusions about the progression from SCD to MCI to be made. Our study is also limited by the absence of an educational background variable. Future research should also include educational background as an explanatory variable, due to its association with olfactory identification and cognitive performance [101,102]. Another limitation is the absence of an Alzheimer’s cohort. Future investigations with longitudinal data should also include conversion to AD. Finally, an important addition to future studies would be neuroimaging measurement. The combination of olfactory, cognitive, and neuroimaging data would offer a more complete photograph of olfaction as a predictor of dementia. In relation to this, Li et al. (2024) [69], in participants who progressed to cognitive decline within a 5-year span, found a relationship between OI, hippocampal cortical volume, and cognitive decline. Results like this support our hypothesis in further studies.

## 5. Conclusions

Our results suggest that combining cognitive assessments with olfactory identification tests could enhance the early detection of individuals at risk. Additionally, incorporating olfactory testing into routine assessments for elderly individuals, especially those reporting subjective cognitive complaints, could provide a non-invasive and cost-effective tool for early diagnosis and intervention. Future research should focus on longitudinal studies to confirm the predictive value of olfactory identification deficits in conjunction with cognitive assessments and to explore the underlying mechanisms linking olfactory function and cognitive impairment.

## Figures and Tables

**Table 1:** Descriptive analysis by cognitive status.

	Cognitively Healthy (CH)	Subjective Cognitive Decline (SCD)	Mild Cognitive Impairment (MCI)			
*Sample Size*	*101*	*47*	*116*			
	Mean (SD) or Count	Mean (SD) or Count	Mean (SD) or Count	F or Chi	*p*	Post Hoc ª
Sex (women)	62	33	77	1.238	0.538	-
Age	69 (7.98)	69.74 (6.83)	66.52 (7.46)	4.448	0.013 *	CH, SCD > MCI
Frequent alcohol consumption	23	5	17	5.227	0.265	-
Identification-Recognition score	13.18 (1.61)	11.23 (3.74)	9.51 (4.44)	29.89	<0.0001 **	CH > SCD > MCI
Identification-Subjective intensity score	6.61 (1.35)	6.05 (2.03)	6.46 (1.91)	1.487	0.228	-
MoCA	28.96 (1.1)	28.67 (1.12)	23.84 (2.07)	305.5	<0.0001 **	CH, SCD > MCI
CVLT—immediate recall score	31.76 (6.6)	30.51 (5.59)	27.74 (6.99)	9.464	0.0001 **	CH, SCD > MCI
CVLT—delayed recall score	7.94 (3.69)	7.74 (2.58)	6.35 (4.39)	4.732	0.009 **	CH, SCD > MCI
Digits—Direct span	5.44 (1.56)	5.17 (1.23)	5.24 (1.54)	0.743	0.477	-
Digits—Inverse span	4.33 (1.65)	4.09 (1.52)	3.76 (1.34)	3.595	0.029 *	CH, SCD > MCI
Cancelation—correct answers	17.31 (4.27)	15.2 (4.93)	17.06 (4.47)	2.867	0.059	-
Cancelation—omissions	8 (9.2)	14.1 (36.94)	5.25 (6.38)	3.534	0.031 *	MCI > CH, SCD
RCFT—immediate recall score	19.17 (6.81)	17.48 (6.62)	16.96 (9.17)	1.968	0.142	-
RCFT—delayed recall score	20.93 (21.87)	25.13 (38.47)	16.81 (7.72)	1.843	0.161	-
Phonological verbal fluency—total score	14.85 (4.71)	14.21 (3.36)	13.14 (4.5)	3.914	0.021 *	CH, SCD > MCI
Semantic verbal fluency—total score	20.6 (6.52)	17.37 (4.74)	17.88 (5.08)	7.844	0.0005 **	CH > SCD, MCI
Vocabulary Test—total score	48.5 (10.98)	45.61 (11.67)	45.21 (10.23)	2.346	0.098	-
TMT A—seconds	47.93 (20.94)	51.53 (14.73)	50.11 (30.51)	0.376	0.687	-
TMT B—seconds	103.9 (55.67)	120.26 (61)	121.69 (91.85)	1.569	0.211	-

* *p* < 0.05; ** *p* < 0.01. ª Post hoc comparisons (*p* < 0.05) under FWER correction.

**Table 2:** Results from multiple linear regression models for cognitive variables.

Cognitive Variables	Predictors	Estimate (b)	Std. Error (SE)	*p*	R^2^	F ª	*p*
CVLT—immediate recall	*Model 1*					11.067	0.001
	Intercept	7.336	1.057	<0.0001 **	0.015		
	Age	−0.033	0.015	0.027 *			
	Sex	0.119	0.241	0.621			
	*Model 2*						
	Intercept	5.206	1.216	<0.0001 **	0.059		
	Age	−0.023	0.015	0.114			
	Sex	0.043	0.237	0.857			
	Olfactory Identification—Recognition	0.125	0.038	0.001 **			
CVLT—delayed recall	*Model 1*					14.581	0.0002 **
	Intercept	14.342	2.258	<0.0001 **	0.052		
	Age	−0.117	0.032	0.0006 **			
	Sex	0.769	0.512	0.134			
	*Model 2*						
	Intercept	10.103	2.461	<0.0001 **	0.104		
	Age	−0.092	0.032	0.003 **			
	Sex	0.752	0.498	0.132			
	Olfactory Identification—Recognition	0.253	0.066	0.0001 **			
Digits—Direct span	*Model 1*					15.646	0.0001 **
	Intercept	10.069	0.778	<0.0001 **	0.129		
	Age	−0.067	0.011	<0.0001 **			
	Sex	−0.314	0.185	0.051			
	*Model 2*						
	Intercept	8.599	0.849	<0.0001 **	0.177		
	Age	−0.059	0.011	<0.0001 **			
	Sex	−0.357	0.231	0.061			
	Olfactory Identification—Recognition	0.09	0.023	0.0001 **			
Digits—Inverse span	*Model 1*					14.581	0.0002 **
	Intercept	7.897	0.864	<0.0001 **	0.068		
	Age	−0.055	0.012	<0.0001 **			
	Sex	−0.191	0.195	0.327			
	*Model 2*						
	Intercept	6.247	0.945	<0.0001 **	0.117		
	Age	−0.047	0.012	0.0001 **			
	Sex	−0.195	0.189	0.306			
	Olfactory Identification—Recognition	0.097	0.025	0.0001 **			
Cancelation—correct answers	*Model 1*					9.414	0.0024 **
	Intercept	29.214	2.892	<0.0001 **	0.071		
	Age	−0.179	0.041	0.0002 **			
	Sex	−0.365	0.662	0.582			
	*Model 2*						
	Intercept	23.817	3.339	<0.0001 **	0.106		
	Age	−0.154	0.041	0.0002 **			
	Sex	−0.557	0.652	0.394			
	Olfactory Identification—Recognition	0.309	0.103	0.003 **			
Cancelation—omissions	*Model 1*					2.329	0.128
	Intercept	−5.657	10.775	0.601	0.001		
	Age	0.194	0.153	0.205			
	Sex	0.808	2.465	0.743			
	*Model 2*						
	Intercept	−15.815	12.638	0.212	0.004		
	Age	0.241	0.255	0.123			
	Sex	0.444	2.469	0.875			
	Olfactory Identification—Recognition	0.598	0.391	0.128			
RCFT—immediate recall score	*Model 1*					11.977	0.0006 **
	Intercept	30.823	4.675	<0.0001 **	0.027		
	Age	−0.172	0.066	0.009			
	Sex	−1.578	1.06	0.138			
	*Model 2*						
	Intercept	20.959	5.379	0.0001	0.074		
	Age	−0.126	0.066	0.058			
	Sex	−1.89	1.038	0.071			
	Olfactory Identification—Recognition	0.568	0.164	0.0006 **			
RCFT—delayed recall score	*Model 1*					2.857	0.092
	Intercept	16.423	14.551	0.26	0.001		
	Age	0.093	0.207	0.652			
	Sex	−4.149	3.262	0.205			
	*Model 2*						
	Intercept	2.397	16.694	0.886	0.008		
	Age	0.149	0.208	0.474			
	Sex	−4.797	3.27	0.144			
	Olfactory Identification—Recognition	0.874	0.518	0.093			
Phonological verbal fluency—total score	*Model 1*					1.399	0.238
	Intercept	13.738	2.634	<0.0001 **	0.001		
	Age	−0.001	0.037	0.973			
	Sex	0.525	0.594	0.378			
	*Model 2*						
	Intercept	12.294	2.901	<0.0001 **	0.001		
	Age	0.005	0.038	0.899			
	Sex	0.544	0.594	0.361			
	Olfactory Identification—Recognition	0.088	0.076	0.248			
Semantic verbal fluency—total score	*Model 1*					18.737	<0.0001 **
	Intercept	36.276	3.23	<0.0001 **	0.101		
	Age	−0.241	0.046	<0.0001 **			
	Sex	−1.648	0.728	0.025 *			
	*Model 2*						
	Intercept	30.038	3.437	<0.0001 **	0.161		
	Age	−0.214	0.044	<0.0001 **			
	Sex	−1.534	0.924	0.051			
	Olfactory Identification—Recognition	0.384	0.088	<0.0001 **			
Vocabulary Test—total score	*Model 1*					7.318	0.007 *
	Intercept	47.359	6.745	<0.0001 **			
	Age	0.027	0.096	0.782			
	Sex	−3.82	1.535	0.013 *			
	*Model 2*						
	Intercept	35.929	7.874	<0.0001 **	0.049		
	Age	0.08	0.097	0.407			
	Sex	−4.338	1.525	0.005 *			
	Olfactory Identification—Recognition	0.577	0.248	0.021 *			
TMT A—seconds	*Model 1*					2.917	0.089
	Intercept	−26.76	13.314	0.055	0.124		
	Age	1.111	0.196	<0.0001 **			
	Sex	1.051	3.15	0.739			
	*Model 2*						
	Intercept	−12.237	16.146	0.449	0.128		
	Age	1.044	0.199	<0.0001 **			
	Sex	1.575	3.151	0.618			
	Olfactory Identification—Recognition	−0.854	0.5	0.089			
TMT B—seconds	*Model 1*					8.637	0.004 *
	Intercept	−77.531	42.478	0.069	0.077		
	Age	2.709	0.604	<0.0001 **			
	Sex	11.171	9.705	0.251			
	*Model 2*						
	Intercept	−1.508	49.116	0.975	0.109		
	Age	2.363	0.605	0.0001 **			
	Sex	13.911	9.585	0.148			
	Olfactory Identification—Recognition	−4.473	1.522	0.004 *			

* *p* < 0.05; ** *p* < 0.0038. ª F test to check if model 2 significantly improves model 1.

**Table 3:** Logistic regression models (baseline or model 1 and definitive or model 2) of CH vs. SCD.

	Estimate	Error	*p*	Sensitivity	Specificity	AUC
*Model 1*						
Intercept	2.235	1.759	0.204	0.59	-	0.5
Age	−0.017	0.024	0.702			
Sex	−0.428	0.383	0.263			
*Model 2*						
Intercept	3.839	2.447	0.117	0.76	0.94	0.66
Age	−0.014	0.027	0.576			
Sex	0.546	0.413	0.186			
Olfactory Identification—Recognition	−0.317	0.083	<0.0001 **			

* *p* < 0.05; ** *p* < 0.017.

**Table 4:** Logistic regression models (baseline or model 1 and definitive or model 2) of CH vs. MCI.

	Estimate	Error	*p*	Sensitivity	Specificity	AUC
*Model 1*						
Intercept	2.051	1343.0	0.127	0.61	0.6	0.6
Age	−0.032	0.019	0.089			
Sex	0.098	0.304	0.746			
*Model 2*						
Intercept	19.822	3.585	<0.0001 **	0.85	0.82	0.83
Age	−0.128	0.031	<0.0001 **			
Sex	0.091	0.447	0.839			
Olfactory Identification—Recognition	−0.967	0.166	<0.0001 **			

* *p* < 0.05; ** *p* < 0.017.

**Table 5:** Logistic regression models (baseline or model 1 and definitive or model 2) of SCD vs. MCI.

	Estimate	Error	*p*	Sensitivity	Specificity	AUC
*Model 1*						
Intercept	4.064	1.727	0.019 *	0.33	0.66	0.5
Age	−0.052	0.025	0.039 *			
Sex	−0.321	0.396	0.419			
*Model 2*						
Intercept	13.883	3.016	<0.0001 **	0.71	0.8	0.73
Age	−0.109	0.033	0.0009 **			
Sex	−0.949	0.491	0.054			
Olfactory Identification—Recognition	−0.479	0.094	<0.0001 **			

* *p* < 0.05; ** *p* < 0.017.

## Data Availability

Data at individual level is available upon request.

## Supplementary Materials

The following supporting information can be downloaded at https://www.mdpi.com/article/10.3390/ejihpe14120196/s1, Table S1: Summary table for the present study’s methods.
