# Supplementary material for: Olfactory Identification as a Biomarker for Cognitive Impairment: Insights from Healthy Aging, Subjective Cognitive Decline, and Mild Cognitive Impairment
Source: Eur J Investig Health Psychol Educ. 2024 Nov 29;14(12):2978–3000. doi: 10.3390/ejihpe14120196 (PMC11675861; doi:10.3390/ejihpe14120196)
Supplement: Supplementary file 1 [file ejihpe-14-00196-s001.zip › ejihpe-3253561-supplementary.pdf]

Table S1. Summary table for the present study methods

Sample	Study design	Measures	Objectives	Statistical analyses
264 participants, from 55 to 90 years, in three cognitive groups: cognitively healthy (CH; n = 101), diagnosed with subjective cognitive decline (SCD; n = 47) and diagnosed with mild cognitive impairment (MCI; n = 116)	Cross-sectional non-experimental design, as no manipulation for independent variables nor random allocation were performed and measures were taken once per participant.	Assessment protocol composed by a screening test of general cognitive status, a questionnaire of cognitive complaints, an olfactory evaluation, and a battery of neuropsychological standardized measures.	Understand the association between OI and cognitive impairment	Two-way between-subject ANOVA model to test the effects of age, cognitive status and the interaction in OI
			Explore the relationship between OI and cognitive domains (memory, executive functioning, attention, executive functioning, visuospatial skills, and language)	Linear regression models to estimate the association between each cognitive variable and OI, with age and sex as covariates
			Study how OI can help to categorize individuals accurately in their respective cognitive group (CH, SCD, MCI)	Logistic regression models to estimate whether OI is a helpful predictor variable to categorize participants in their respective cognitive groups (CH vs SCD, CH vs MCI and SCD vs MCI).
